# Interplay Between m^6^A RNA Methylation and Regulation of Metabolism in Cancer

**DOI:** 10.3389/fcell.2022.813581

**Published:** 2022-02-03

**Authors:** Youchaou Mobet, Xiaoyi Liu, Tao Liu, Jianhua Yu, Ping Yi

**Affiliations:** ^1^ Department of Obstetrics and Gynecology, The Third Affiliated Hospital of Chongqing Medical University, Chongqing, China; ^2^ Laboratory of Biochemistry, Faculty of Science, University of Douala, Douala, Cameroon; ^3^ Department of Hematology and Hematopoietic Cell Transplantation, City of Hope National Medical Center, Los Angeles, CA, United States; ^4^ Hematologic Malignancies and Stem Cell Transplantation Institute, City of Hope National Medical Center, Los Angeles, CA, United States; ^5^ Comprehensive Cancer Center, City of Hope, Los Angeles, CA, United States

**Keywords:** M^6^A, methylation, reprogramming, metabolism, metabolite, oncogenic, cancer

## Abstract

Methylation of adenosine in RNA to N6-methyladenosine (m^6^A) is widespread in eukaryotic cells with his integral RNA regulation. This dynamic process is regulated by methylases (editors/writers), demethylases (remover*/*erasers), and proteins that recognize methylation (effectors/readers). It is now evident that m^6^A is involved in the proliferation and metastasis of cancer cells, for instance, altering cancer cell metabolism. Thus, determining how m^6^A dysregulates metabolic pathways could provide potential targets for cancer therapy or early diagnosis. This review focuses on the link between the m^6^A modification and the reprogramming of metabolism in cancer. We hypothesize that m^6^A modification could dysregulate the expression of glucose, lipid, amino acid metabolism, and other metabolites or building blocks of cells by adaptation to the hypoxic tumor microenvironment, an increase in glycolysis, mitochondrial dysfunction, and abnormal expression of metabolic enzymes, metabolic receptors, transcription factors as well as oncogenic signaling pathways in both hematological malignancies and solid tumors. These metabolism abnormalities caused by m^6^A’s modification may affect the metabolic reprogramming of cancer cells and then increase cell proliferation, tumor initiation, and metastasis. We conclude that focusing on m^6^A could provide new directions in searching for novel therapeutic and diagnostic targets for the early detection and treatment of many cancers.

## Introduction

Adenosine methylation is the most common modification of RNA in eukaryotes. The methyl group is attached to the nitrogen-6 position of adenosine, creating N6-methyladenosine or m^6^A (Wang et al., 2017). This modification is highly dynamic and reversible, as it involves enzymes that methylate adenosine (writers), remove methylation (erasers), or recognize it (readers) (Chen et al., 2019b). Moreover, the m^6^A modification is integral to the regulation of RNA, as it affects mRNA processing, mRNA translation, mRNA decay, mRNA export to the cytoplasm, and miRNA maturation (Roundtree et al., 2017a). In the past several years, compelling evidence has witnessed the implication of m^6^A in RNA modification. Recent work has uncovered that m^6^A plays an important role in gene expression regulation emerged as critical post-transcriptional modifications. Currently, Shi et al., (2019) review advances progress in understanding the mechanisms which specific cellular contexts and molecular function of N6-methyladenosine and highlight the importance of RNA modification regulation, including mRNA, tRNA, rRNA, and other non-coding RNA. They conclude that the recent biological outcome of m^6^A methylation could be promising for translational medicine. Previously, the roles of m^6^A modifications in modulating gene expression throughout cell differentiation and animal development were reviewed by Frye et al., (2018). Their study illustrates that m^6^A methylation plays a critical role by regulating various aspects of RNA metabolism, physiological processes, and stress response (Frye et al., 2018). More interestingly, others recent evidence indicates that the modification of m^6^A also regulates physiology and metabolism in tumors (Faubert et al., 2017; Choe et al., 2018).

Metabolic reprogramming in cancer cells was discovered to promote tumorigenesis (Frezza, 2020). Biochemical and molecular studies have suggested several possible mechanisms for its evolution during cancer development (Hanahan and Weinberg, 2011). Recently, m^6^A’s function in oncology and its involvement in the regulation of cancer metabolism has received growing attention. As a result, our understanding of the metabolic mechanisms regulated by the m^6^A’s modification in carcinogenesis and their potential therapeutic implications have progressed significantly.

Interestingly, m^6^A can act as a suppressor or promoter in the proliferation (Liu et al., 2018; Shen, 2020), differentiation (Chen et al., 2019a), and metastasis of tumor cells (Ma et al., 2017) in various cancers. It also appears to reprogram cancer cell metabolism (Shen et al., 2020), as it can regulate metabolic enzymes, transporters, pathways, and transcription factors that promote cancer progression (Li et al., 2020c; Chen et al., 2021a). Here, we discuss the current understanding of how the m^6^A modification affects cancer metabolism and the potential for regulating it to provide new targets for cancer therapy.

## M^6^A Regulation

Modification of m^6^A is regulated by: methyltransferases that catalyze methylation (writers), demethylases that remove (erasers) the methyl group from m^6^A, then m^6^A recognition proteins (readers) recognize the modification (Lewis et al., 2017). Interestingly, m^6^A methyltransferase, m^6^A demethylases, and m^6^A recognition proteins play essential roles in gene regulation.

### m^6^A Methyltransferase

Methyltransferase-like3 (METTL3) and Methyltransferase-like14 (METTL14) are the critical components of the m^6^A methyltransferase complex (MTC). These two methyltransferases colocalize in the nucleus (Liu et al., 2014), forming a heterodimer. METTL3 transfers the methyl of the S-adenosyl methionine (SAM) to produce S-adenosyl homocysteine (SAH) and leads to global miRNA downregulation. By binding with eIF3h in the cytoplasm, METTL3 can also promote oncogenic mRNAs translation (Choe et al., 2018). METTL3 could be modulated through post-transcriptional modifications, affecting protein stability, localization, writer complex formation, and writer catalytic activity (Shi et al., 2019). In comparison, METTL14 identifies specific RNA sequences as a target and stabilizes the structure of MTC (Liu et al., 2014; Lin et al., 2016; Sledz and Jinek, 2016). For example, METTL14 can methylate target miRNA by cooperating with HNRNPA2B1 and DGCR8, promoting miRNA maturation (Alarcon et al., 2015).

Wilms’ tumor associating protein (WTAP), another writer protein, plays a role in localizing the methylase complex in the nucleus by interaction with heterodimer (Ping et al., 2014; Knuckles et al., 2018). Recently, other components, such as HAKAI, ZC3H13, and VIRMA/KIAA1429, have been identified to interact with other parts of the MTC (Yue et al., 2018), while ZCCHC4 is a ribosomal RNA-28S methyltransferase (Ma et al., 2019). Other methyltransferase components like METTL5 have been found to be independent m^6^A writers. It catalyzes the attachment of m^6^A onto specific structure RNAs, including U6-small nuclear RNA (snRNA), 18S rRNA, and 28S rRNA (Wang et al., 2017; Ignatova et al., 2020). METTL16 catalyzes m^6^A of the U6- spliceosomal small nuclear RNA and MAT2A 3′-UTR mRNA (Pendleton et al., 2017).

### m^6^A Demethylases

The m^6^A remover proteins erase the m^6^A modification by increasing the level of iron ferrous (Fe^2+^) (co-factor) and α-ketoglutarate (co-substrate) dependent oxygenase family (Fedeles et al., 2015). Two erasers that catalyze m^6^A demethylation ALKB homolog 5 (ALKBH5) and fat mass and obesity-associated protein (FTO) can recognize adenine and cytosine methylation in RNA (Fu et al., 2013). ALKBH5 and FTO are members of the Fe^2+^/α-ketoglutarate-dependent dioxygenases family. The first RNA demethylase identified FTO was reported to remove the methyl group of N6 - methyladenosine (m^6^A) in RNA. m^6^A erasers may exhibit different expression levels, post-translational modifications, and cellular localization, depending on cell types. For instance, m^6^A demethylase FTO is predominantly nucleus localized and regulates 5–10% of total mRNA m^6^A demethylation (Wei et al., 2018). In contrast, FTO is also highly abundant in the cell cytoplasm and can mediate up to 40% m^6^A demethylation of total mRNA in certain leukemia cells (Shi et al., 2019). Additionally, FTO regulates alternative splicing via m6A by interacting with Serine-rich splicing factor 2(SRSF2) (Bartosovic et al., 2017). Interestingly, FTO may control metabolic disorders. ALKBH5 another m^6^A demethylase, affects mRNA export and processing factors (Zheng et al., 2013). ALKB homolog 3 (ALKBH3) was found to demethylate only tRNAs (Ueda et al., 2017; Yang et al., 2020).

### m^6^A Recognition

The m^6^A recognition proteins (readers) control the destiny of RNAs that have been modified. Readers/effectors are distributed in the nucleus and cytoplasm, indicating their functional diversity. While writers and erasers carry out methylation and demethylation, the readers determine the functional consequences of modification. m^6^A recognition proteins characterization has provided valuable insights into the molecular mechanisms of the m^6^A-mediated post-transcriptional gene regulation (Shi et al., 2019). Furthermore, RNA binding proteins (RBPs) could regulate the interactions between m^6^A effectors and RNA substrates.

YTHDF1/2/3 and YTHDC1 recognize the m^6^A change and alter mRNA’s splicing, translation, and decay (Xu et al., 2014; Wu et al., 2017a). Intriguingly, these proteins also play crucial roles in mRNA metabolism (Wang et al., 2015). For instance, YTHDF1 binds to mRNA, including eukaryotic translation initiation factor 3 (eIF3) and poly-A- binding protein (PABP) complex to promote RNA translation (Wang et al., 2015). YTHDF2 recognizes mRNAs not destined for translation, accelerating their destruction. Interestingly, it identifies specific m^6^A -modified binds to CCR4-NOT transcription complex subunit 1 (CNOT1). However, it recruits the CCR4-NOT complex of the m^6^A -tagged RNA P-body to promote its destruction (Du et al., 2016). YTHDF3 by interaction with YTHDF1 accelerates mRNA translation, affecting YTHDF2-mediated degradation of mRNAs labeled with m^6^A (Li et al., 2017a).

YTHDC1 mediates mRNA export marked with m^6^A by interaction with the nuclear export adaptor protein SRSF3 (Roundtree et al., 2017b). Importantly, YTHDC1 regulates splicing events by inhibiting SRSF10 or activating splicing factor SRSF3. In conjunction with nuclear RNA export factor 1 (NXF1), YTHDC1 can also mediate mRNA export to the cytoplasm. Unlike the rest of the family, YTHDC2, an RNA helicase. Its helicase domain contributes to RNA binding (Hsu et al., 2017). Significantly, YTHDC2 and YTHDF3 can facilitate RNA degradation or enhance RNA translation depending on the context (Shi et al., 2017).

hnRNPs, another m^6^A recognition family, is localized in the nucleus where heterogeneous nuclear ribonucleoprotein C (hnRNPC) can bind with nascent RNA transcripts and control their processing (Alarcon et al., 2015). For instance, the lncRNA MALAT1 facilitates a change in the m^6^A site for recognition and binding by hnRNPC (Liu et al., 2015). Interestingly, m^6^A regulates RNA binding motifs (RBMs) accessibility by altering mRNA and long noncoding RNA (lncRNA) structure to promote hnRNPC interaction. These changes influence RNA-protein interactions in human cells. This mechanism is called the “m^6^A -switch” (Liu et al., 2015). hnRNPC-binding regulated by the m^6^A -switch regulates RNA alternative splicing, indicating that the switch helps regulate gene expression and RNA maturation (Liu et al., 2015).

Another component of the m^6^A recognition family, Heterogeneous nuclear ribonucleoprotein A2B1 (hnRNPA2B1), regulates RNA alternative splicing and microRNA processing (Alarcon et al., 2015; Liu et al., 2015). Further, it interacts with DiGeorge syndrome critical region gene 8 (DGCR8) for miRNA maturation and recognizes the m^6^A signals of microRNA (Zhao et al., 2017). Eukaryotic initiation factor 3 (eIF3), another effector/reader, could initiate protein translation in a cap on its 5′-UTR (Meyer et al., 2015). In conjunction with Hu antigen R (HuR), these proteins recognize m^6^A’s modification and stabilize their RNA transcripts (Meyer et al., 2015).

**FIGURE 1:**
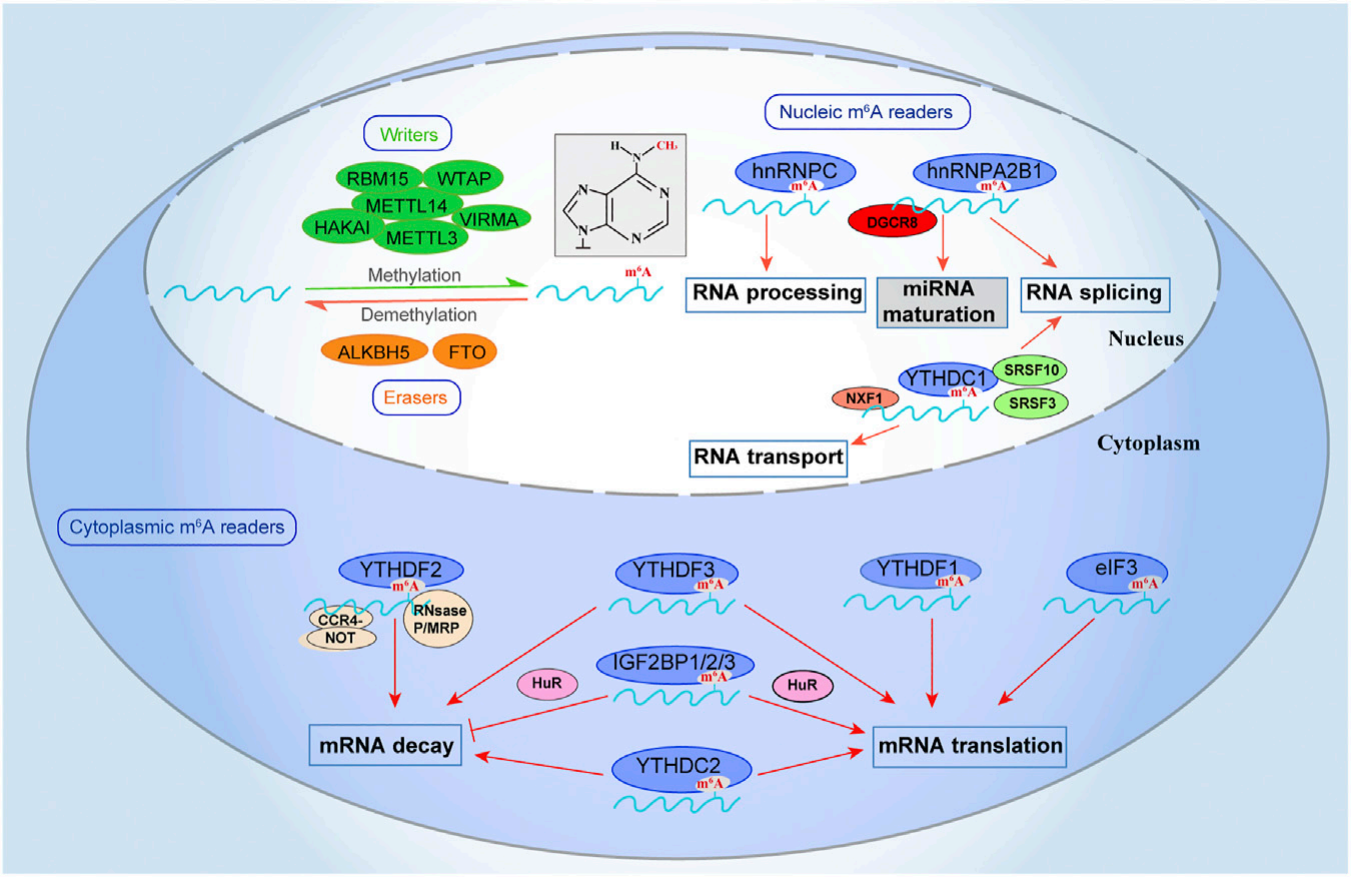
m^6^A-mediated RNA regulation. The m^6^A modification is integral to the regulation of RNA. m^6^A can be installed by “writers” (METTL3/14, WTAP, RBM15, VIRMA, and HAKAI), removed by “erasers” (FTO and ALKBH5), and recognized by “readers” (YTHDF1/2/3, YTHDC1/2, IGF2BP1/2/3, eIF3, and HNRNPC/A2B1). m^6^A methyltransferases (writers) catalyze methylation while the m^6^A demethylases (erasers) remove the methyl in m^6^A. The m^6^A recognition (readers) proteins bind the m^6^A modified transcripts and determine their fate. The modification of “writers,” “erasers,” and “readers” proteins affect RNA processing, including RNA splicing, mRNA translation, mRNA decay, mRNA export to the cytoplasm, and miRNA maturation.

Insulin growth factor-2 binding proteins 1, 2, and 3 (IGF2BP 1/2/3) were identified as another m^6^A recognition. After co-localizing with HuR, these proteins protect mRNA decay and enhance mRNA translation (Huang et al., 2018). These findings demonstrated that m^6^A methyltransferases (editors/writers) and m^6^A demethylases (remover*/*erasers) cooperate to modulate the distribution of m^6^A on RNA by adding (writer) or removing (erasers) the methyl. While the m^6^A recognition (effectors/readers) proteins recognize the m^6^A modified transcripts and determine their fate regulate functions (Figure 1).

## M^6^A Regulates Cancer Metabolism

Cancer cells need abundant energy and raw materials to grow and divide; therefore, they substantially alter their metabolic pathways (Hanahan and Weinberg, 2011; Li and Zhang, 2016). Importantly, biochemical and molecular studies suggest several possible mechanisms for the evolution of aberrant metabolism during cancer development (Hanahan and Weinberg, 2011). For example, proliferating cancer cells can enhance the synthesis of glucose of carbohydrates, lipids, and proteins to obtain an ample and uninterrupted supply of molecules needed for biosynthesis (Khan et al., 2020). Moreover, most cancer cells depend on aerobic glycolysis rather than the TCA cycle (Vander Heiden et al., 2009). The preference for glycolysis over mitochondrial oxidative phosphorylation seems to be a hallmark of cancer cells (Garber, 2006).

**FIGURE 2:**
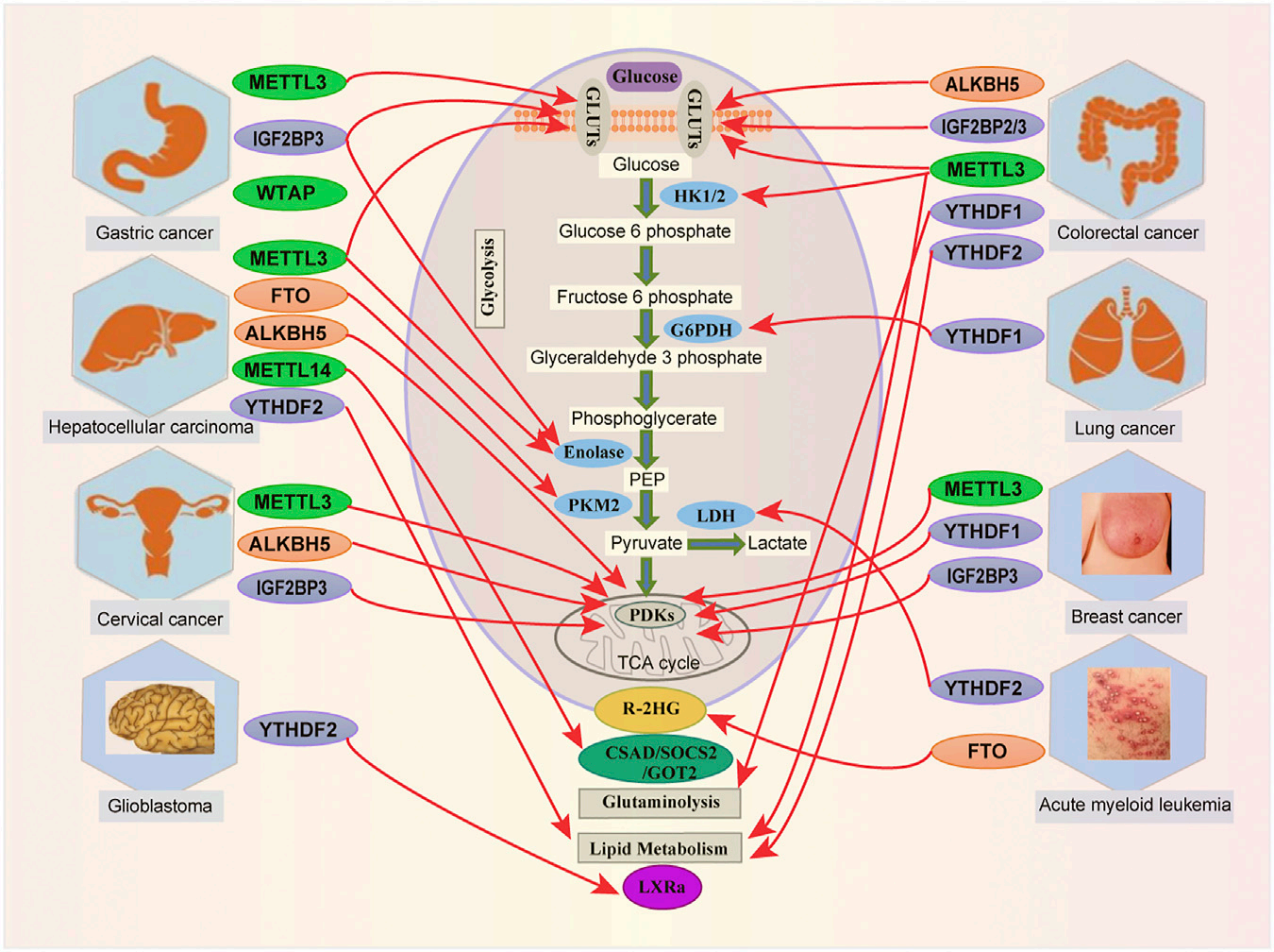
Links between m^6^A modification and metabolites in human cancer. m^6^A RNA modification by targeting metabolic pathways is involved in various tumorigenesis, including Acute Myeloid Leukemia (AML), Breast Cancer (BC), Cervical Cancer (CC), Colorectal Cancer (CRC), Glioblastoma (GBM), Hepatocellular Carcinoma (HCC), Gastric Cancer (GC) and Lung Cancer (LC).

However, aerobic glycolysis transports chemical generates ATP. This ATP and its breakdown product adenosine are widespread throughout the body, and both have been shown to regulate cell proliferation and differentiation. Therefore, metabolic reprogramming is widely utilized during oncogenesis, and the m^6^A modification can regulate metabolism in cancer progression (Figure 2).

### m^6^A Regulates Glucose Metabolism

Glucose, an essential nutrient in blood, is the main energy source for cells (Shaw, 2006). However, several studies have found that hyperglycemia increases the overall risk of cancer (Stattin et al., 2007). Cancer cells enhanced glucose uptake has also been implicated in metastasis and poor prognosis (Macheda et al., 2005). Aerobic glycolysis in cancer can increase the m^6^A modification genes associated with glycolysis (Fry et al., 2017).

Recent evidence demonstrated that cancer reprograms glucose metabolism (Li et al., 2020c); thus, aerobic glycolysis exemplifies an evolutionary change in cancer cells. Not surprisingly, glycolytic transporters like glucose transporter (GLUT), glycolytic enzymes such as pyruvate kinase isozyme M1/2 (PKM1/2), pyruvate dehydrogenase kinase (PDK), lactate dehydrogenase (LDH), and hexokinase (HK) is important targets to understand cancer metabolism (Doherty and Cleveland, 2013; Viale et al., 2014). The relationship between m^6^A and glucose metabolism is crucial for understanding cancer progression because glucose is the most important metabolite associated with many enzymes and transporters. Additionally, glycolysis is an essential pathway involved in cancer progression, metastasis, and chemotherapy resistance (Ganapathy-Kanniappan and Geschwind, 2013).

In Colorectal Cancer (CRC), the METTL3-HK2/GLUT1-MYC-IGF2BP is involved in cells proliferation and metastasis (Shen et al., 2020; Xiang et al., 2020; Chen et al., 2021a). Hexokinase (HK) catalyzed the first step of anaerobic glycolysis and oxidative phosphorylation, which converts glucose to glucose 6-phosphate (G6P) (Wilson, 2003). Many investigations reveal the implication of HK in cancers. For instance, HK2 bound to mitochondria enable cancer cells to become more glycolytic (Chen et al., 2014; Wu et al., 2017b). GLUT1, a glucose transporter, mediates the first step of glucose inside cells (Cheeseman, 2008). Overexpression of GLUTs facilitates glucose consumption in cancer progression (Ancey et al., 2018). METTL3 stabilizes GLUT1 and HK2 mRNA in colorectal cancer by directly interacting with the 3′ UTR mRNA of GLUT1 and the 5'/3′-UTRs mRNA of HK2. This enhanced HK2 and GLUT1 expression, promoting CRC progression (Shen et al., 2020). One recent study established that METTL3 enhanced CRC growth and identified GLUT1-mTORC1 as the primary target of METTL3 in that disease (Chen et al., 2021a). More interestingly, METTL3 induced GLUT1 translation in m^6^A to promote glucose uptake and lactate production, leading to mTORC1 activation. These findings indicate that METTL3 promotes CRC *via* the m^6^A-mediated GLUT1-mTORC1 signaling activation.

In Cervical Cancer, (Li et al., 2020c) demonstrated that m^6^A regulates glycolysis in cancer cells through pyruvate dehydrogenase kinase 4 (PDK4). PDKs are the gatekeeper enzymes involved in altered glucose metabolism in tumors (Patel and Korotchkina, 2006; Devedjiev et al., 2007). They are remarkably overexpressed in multiple human tumor samples. Among them, PDK4 was noticed as one critical metabolic factor of metabolism control because it can divert carbon flux from oxidative phosphorylation into glycolysis (OXPHOS) (Stacpoole, 2017). According to Li and collaborators, the extracellular acidification rate (ECAR) was decreased in Mettl3^Mut/-^ HeLa cells, While the oxygen consumption rate (OCR) was increased (Li et al., 2020c), demonstrating that METTL3 promotes glycolysis. Additionally, PDK4 can reverse lactate production level, glucose consumption, and ATP rate in Mettl3-depleted cells. More importantly, overexpression of PDK4 to an endogenous level attenuated the metabolic phenotypes of SiHa cells that had lost METTL3. Also, overexpression of ALKBH5 suppressed PDK4 expression in HeLa cells (Li et al., 2020c). Moreover, compared with negative control samples, IGF2BP3 and YTHDF1 were significantly higher in cervical cancer samples (Li et al., 2020c). Li et al. (2020b) further determined whether the m^6^A modification can regulate PDK4 expression in addition to affecting the stability of the kinase’s mRNA. Modifying the PDK4 mRNA at its 5′-UTR by m^6^A positively regulated its elongation during translation and the stability of its mRNA because m^6^A is bound to the YTHDF1 and IGF2BP3 (Li et al., 2020c). In HeLa cells, IGF2BP3 inhibition can suppress PDK4 expression and alter the suppressive effect of METTL3 on PDK4 expression (Li et al., 2020c). More interestingly, YTHDF1 and IGF2BP3-targeting PDK4 with d m^6^A CRISPR significantly downregulated PDK4 mRNA and protein levels (Li et al., 2020c). Thus, targeting m^6^A on PDK4 mRNA with dm6ACRISPR appears to regulate glycolysis and ATP generation in cancer (Li et al., 2020c). These studies suggest that PDK4 is a critical metabolic agent of glycolysis and ATP generation regulated by m^6^A in cervical cancer progression.

In Hepatocellular Carcinoma (HCC), hepatic FTO helps regulate the expression of the gluconeogenic gene. Recent evidence indicates that demethylation of m^6^A by FTO affects glucose metabolism *via* hepatic gluconeogenesis (Shen et al., 2015). On the other hand, the FTO level may be affected by insulin in HCC (Mizuno et al., 2017). Pyruvate kinase isozymes M1 (PKM1) and M2 (PKM2) are glycolytic enzymes (Doherty and Cleveland, 2013). They mediate the final steps of glycolysis by dephosphorylation of phosphoenolpyruvate (PEP), producing pyruvate and ATP. According to (Li et al., 2019a), FTO promotes HCC tumorigenesis by demethylating m^6^A on PKM2 mRNA. This demethylation accelerates translation, leading to tumorigenesis in HCC (Li et al., 2019a). The demethylation of PKM2 mRNA by FTO suggests that FTO also regulates the expression of PKM2. Knocking down FTO repressed HCC progression (Li et al., 2019a). This finding revealed that FTO could demethylate PKM2 mRNA, thereby upregulating the kinase’s expression. Upregulated PDK4 was found to reduce hepatic chemotherapy-induced colorectal liver metastasis (Strowitzki et al., 2019). PDK4 collaborates with METTL3 to induce proliferation and hepatic chemosensitivity cancer cells (Li et al., 2020c). Regarding the link between PDK4 and m^6^A, Li and collaborators found that m^6^A -PDK4 plays an essential role in liver cancer progression. Consistent with this finding, knocking down METTL3 inhibited PDK4 antibodies in Huh7 cells. Moreover, overexpression of the demethylase ALKBH5 (another m^6^A eraser) decreased glucose, lactate, and ATP abundance in Huh7 HCC cells (Li et al., 2020c). Li and collaborators also provided evidence that METTL3 regulates glycolytic activity in HCC. Downregulation of METTL3 cooperates with the 2-deoxyglucose (2-DG) to inhibit HCC proliferation*,* suggesting that suppressing glycolysis by inhibiting METTL3 might be a potential strategy for treating HCC (Lin et al., 2020).

In Acute Myeloid Leukemia (AML), *α*-ketoglutarate, produced by isocitrate dehydrogenase in the TCA cycle, interacts with m^6^A demethylase proteins (Losman et al., 2020). R-2HG (R-2-hydroxyglutarate) inhibited FTO activity by stimulating the modification of m^6^A -RNA in cells. Moreover, through targeting the FTO/MYC/CEBPA axis, R-2HG inhibited the proliferation of leukemia cells (Su et al., 2018). It was reported that knocking down FTO or LDHB (lactate dehydrogenase B) inhibits R-2HG in leukemia cells (Qing et al., 2021). Additionally, R-2HG abrogated FTO/m^6^A/YTHDF2-mediated upregulation of LDHB, suppressing aerobic glycolysis (Qing et al., 2021). These findings show that R-2HG attenuates aerobic glycolysis by inhibiting FTO in leukemia cells. Lactate dehydrogenase (LDH) converts pyruvate to lactate, and this enzyme is frequently upregulated in multiple cancers (Wang et al., 2012). Lactate, ketone, and pyruvate are monocarboxylates that play essential roles in cancer metabolism (Halestrap, 2013).

In Gastric Cancer (GC), overexpression of METTL3 (a writer) promoted metastasis to the liver *in vitro* and *in vivo,* and it also stimulated the modification of adenosine to m^6^A, enhancing mRNA stability (Wang et al., 2020c). Tumor angiogenesis was promoted by Hepatoma-derived growth factor (HDGF) upregulation, while nuclear HDGF activated GLUT4 and ENO2 expression and increased metastasis in GC cells (Wang et al., 2020c). WTAP (a writer) promoted GC cell proliferation and glycolytic capacity and enhanced HK2 expression through interacting with the m^6^A modified 3′-UTR of HK2 mRNA (Yu et al., 2021).

In Glioblastoma (GBM), Li et al. recently showed that long noncoding RNA just proximal to X-inactive specific transcript (JPX) interacted with N6-methyladenosine (m^6^A) demethylase FTO and enhanced FTO-mediated PDK1 mRNA demethylation. Additionally, JPX exerted its GBM-promotion effects through the FTO/PDK1 axis (Li et al., 2021). These outcomes reveal the critical role of JPX in promoting GBM aerobic glycolysis-m^6^A demethylase FTO.

In Lung Cancer (LC), YTHDF2 expression is increased in tumor tissues, promoted proliferation, and bound to 3′-UTR of 6-phosphogluconate dehydrogenase (G6PD) mRNA (Sheng et al., 2020). This binding facilitates G6PD mRNA translation in LC and promotes tumorigenesis. Recently, Yang and collaborators showed that FTO is declined in lung adenocarcinoma, which correlates with poor patient overall survival. Moreover, downregulated FTO expression enhanced m^6^A levels in mRNAs of genes involved in metabolic pathways such as MYC (Yang et al., 2021). Interestingly, the enhanced levels recruited the binding of YTHDF1, which promoted the translation of MYC mRNA and increased glycolysis and cancer progression (Yang et al., 2021).

In Breast Cancer (BC), METTL3 overexpression enhanced the PDK4 protein expression in breast cancer cells (Li et al., 2020c). Interestingly, the m^6^A -modified 5′-UTR of PDK4 regulated the kinase’s elongation during translation and the stability of its mRNA through interaction with YTHDF1 and IGF2BP3. Further, clinical data confirm that m6A/PDK4 is implicated in breast cancer progression (Li et al., 2020c). These findings suggest that proteins associated with m^6^A regulate glycolysis in breast cancer cells.

### m^6^A Regulates Lipid Metabolism

Recently, elevated lipid levels were recognized as an important aberration of cancer metabolism (Swierczynski et al., 2014). Moreover, previous studies have noticed that lipid metabolism is reprogrammed in tumors (Schulze and Harris, 2012; Nath and Chan, 2016). Dysregulation of lipid metabolism is an essential feature of cancer cells (Murai, 2015; Gaida et al., 2016).

There is also a link between m^6^A proteins and lipid metabolism in cancer. After observing that knocking down METTL3 and YTHDF2 decreased lipid accumulation in hepatocellular carcinoma cells, Zhong et al. (2018) proposed that the presence of m^6^A in mRNA mediates crosstalk between the circadian clock and lipid metabolism (Zhong et al., 2018). Kang et al. (2018) showed that FTO increased triglyceride (TG) deposition and decreased mitochondrial content. FTO regulates lipid metabolism in hepatocytes by modulating RNA m^6^A levels (Kang et al., 2018). These studies revealed that FTO’s demethylating is an important actor in the lipid metabolism of hepatocytes. By linking the epigenetic modification of RNA with fat deposition, they suggested a new m^6^A target for regulating hepatic fat metabolism (Kang et al., 2018). FTO overexpression in HepG2 cells also reduced m^6^A levels, enhancing stearoyl CoA desaturase (SCD), monoacylglycerol O acyltransferase 1 (MOGAT1), and fatty acid synthase (FAS), which contribute to cell growth (Kang et al., 2018). Numerous studies demonstrated that METTL3-mediated m^6^A modification and inhibition of mRNA decay promoted the miR-3619-5p/HDGF axis, enhancing lipogenesis in Hepatocellular Carcinoma (Zhong et al., 2019; Zuo et al., 2020).

In GBM, Fang et al. (2021) recently showed that YTHDF2 facilitates m^6^A -dependent mRNA decay, impacting glioma patients’ survival. Moreover, YTHDF2 inhibited cholesterol homeostasis in GBM cells. These outcomes highlight the critical function of YTHDF2 regulated cholesterol homeostasis in GBM (Fang et al., 2021). Other reported studies showed that YTHDF2 could also regulate lipogenic genes, including acetyl CoA carboxylase 1 (ACC1), fatty acid synthase (FAS), and stearoyl-CoA desaturase 1(SCD1), to decrease their mRNA stability (Zhou et al., 2021).

As a lipid, sphingolipids also regulate cancer proliferation, migration, invasion, and metastasis. Among this class of lipid, delta 4 desaturase sphingolipid 2 (DEGS2) catalyzes the conversion of dhCers to phytoceramides (Casasampere et al., 2016). Recently, Guo and collaborators found the role of m^6^A modification on DEGS2 in colorectal cancer and suggested that inhibited m^6^A promotes DEGS2 expression and dysregulated lipid metabolites, contributing to colorectal cancer (Guo et al., 2021). Furthermore, overexpression of DEGS2 promoted cell growth, while depletion of DEGS2 inhibited cell growth (Casasampere et al., 2016). Regarding the molecular mechanism, Guo and collaborators found that METTL3 depletion promoted the DEGS2 mRNA, increased DEGS2 expression in HCT116 cells, suggesting that METTL3 is essential for the stability and translation of DEGS2. YTHDF2 knockdown induced the level of DEGS2 mRNA expression, meaning that YTHDF2 contributes to the DEGS2 mRNA decay (Guo et al., 2021). Collectively, this recent evidence suggests that m^6^A regulates lipid metabolism in cancer.

### m^6^A Regulates Amino Acid Metabolism

To proliferate, cancer cells need large amounts of amino acids (Sivanand and Vander Heiden, 2020), which are essential building blocks of proteins (Murugan, 2019; Vettore et al., 2020). Moreover, there is much evidence for specific degradation in amino acid metabolism in cancers (Li and Zhang, 2016). Glutamine, which regulates the expression of many genes related to metabolism (Curi et al., 2005), is carried into cancer cells by multiple transporters, such as Na + **-**coupled neutral amino acid transporters (SNATs) and Na + -dependent transporters (Kandasamy et al., 2018).

To renew the TCA cycle, many tumor cells highly need glutamine (Matés et al., 2013). Glutamate dehydrogenase (GLUD1) and transaminases can transform glutamine to *α*-KG to reconstruct the TCA cycle (Vander Heiden et al., 2009). FTO and ALKBH5 were identified as *α*-KG-dependent dioxygenases (Zhu et al., 2020). METTL14 may promote HCC progression by modulating m^6^A -regulated genes, including glutamic oxaloacetic transaminase 2 (GOT2), cysteine sulfonic acid decarboxylase (CSAD), and suppressor of cytokine signaling 2 (SOCS2) (Li et al., 2020b). In colon cancer, Chen and collaborators demonstrated YTHDF1-mediated as a positive association between glutamine metabolism and cisplatin resistance (Chen et al., 2021b).

Recently, reports have indicated that AMP-activated protein kinase (AMPK) could act as a beneficial target for treating cancer patients (Wang et al., 2016b). AMPK can act to inhibit tumorigenesis through the regulation of cell proliferation. AMP-activated protein kinase-alpha2 (AMPKα2) was inversely correlated with FTO (Wang et al., 2016a). FTO is upregulated in colorectal cancer and interacts with MYC to accelerate cell proliferation and migration (Zou et al., 2019). In colorectal cancer, Yue and collaborators reveal that AMPKα2 inhibits CRC cell growth and promotes apoptosis through altering FTO (Yue et al., 2020). More interestingly, miR-96 could retard cancerogenesis by inactivating the FTO-mediated MYC AMPKα2-dependent manner in CRC cells (Yue et al., 2020). Together, these findings elucidate links between m^6^A and metabolic changes in cancers (Table 1 and Figure 2).

**TABLE 1:** Regulation of metabolites by m^6^A associated proteins in cancer.

Metabolic pathways	Metabolites/Enzymes/Signaling pathways	m^6^A associated proteins	Cancer type	Role in cancer	References
Glycolysis	GLUT1-mTORC1	METTL3	CRC	Oncogene	Chen et al. (2021a)
	GLUT1	METTL3/IGF2BP2/3	CRC	Oncogene	Shen et al. (2020)
	PDK4	METTL3	Breast cancer	Oncogene	Li et al. (2020c)
	HK2	METTL3	CRC	Oncogene	Shen et al. (2020)
	GLUT4/Enolase	METTL3	Liver cancer	Oncogene	Wang et al. (2020c)
	GLUT4/HDGF/ENO2	METTL3/IGF2BP3	Gastric cancer	Oncogene	Wang et al. (2020c)
	PDK4	METTL3/IGF2BP3/ALKBH5	Cervical cancer	Oncogene	Li et al. (2020c)
	MYC	FTO/YTHDF1	Lung cancer	Oncogene	Yang et al. (2021)
	PDK4	METTL3	Liver cancer	Oncogene	Li et al. (2020c)
	HK2	WTAP	Gastric cancer	Oncogene	Yu et al. (2021)
	PDK4	YTHDF1/IGF2BP3	Breast cancer	Oncogene	Li et al. (2020c)
	PKM2	FTO	HCC	Oncogene	Li et al. (2019a)
	PDK4	ALKBH5	Cervical cancer	Oncogene	Li et al. (2020c)
	PDK4	ALKBH5	HCC	Oncogene	Li et al. (2020c)
	GLUT1	ALKBH5	CRC	Oncogene	Shen et al. (2020)
	G6PD	YTHDF2	Lung cancer	Oncogene	Sheng et al. (2020)
	2-deoxyglucose	METTL3	HCC	Oncogene	Lin et al. (2020)
	MYC	METTL3	CRC	Oncogene	Lin et al. (2020)
	LDHB	YTHDF2	AML	Oncogene	Qing et al. (2021)
Lipid metabolism	Lipid	METTL3/YTHDF2	Liver cancer	Oncogene	Zhong et al. (2018)
	Cholesterol	YTHDF2	Glioblastoma cancer	Oncogene	Fang et al. (2021)
	Triglyceride	METTL3	Liver cancer	Oncogene	Kang et al. (2018)
	Sphingolipid (DEGS2)	METTL3/YTHDF2	CRC	Oncogene	Guo et al. (2021)
Glutaminolysis	R-2HG-MYC	FTO	Leukemia	Oncogene	Su et al. (2018)
	CSAD/GOT2/SOCS2	METTL14	HCC	Oncogene	Li et al. (2020b)
	R-2HG	FTO	AML	Oncogene	Qing et al. (2021)
	Glutamine	YTHDF1	Colon cancer	Oncogene	Chen et al. (2021b)
Other metabolic	Iron and ferritin metabolism	YTHDF1	HPSCC	Oncogene	Ye et al. (2020)

### Other Metabolic Processes Regulated by m^6^A in Cancer

Emerging evidence demonstrates that m^6^A can also regulate metabolic processes in carcinogenesis that do not involve glucose, lipids, or amino acids. For example, iron metabolism plays a key role in tumorigenesis (Jung et al., 2019). Therefore, pathways that acquire, export, or store iron are often perturbed in cancer (Jung et al., 2019). The tumor microenvironment exerts selective pressure that renders the cancer cells adopt altered metabolism, supporting these cells’ energy and metabolic demands, thereby facilitating tumor growth. Recent evidence showed that tumor-associated macrophages (TAMs) could provide iron to impact metabolism within the tumor microenvironment. When Ye and collaborators evaluated the correlation between the m^6^A modification and iron metabolism, they found that YTHDF1 regulates growth and iron metabolism in hypopharyngeal squamous cell carcinoma (HPSCC) (Ye et al., 2020). YTHDF1 was also associated with intratumoral iron and ferritin levels in hypopharyngeal squamous cell carcinoma (HPSCC) patients. They further demonstrated that HPSCC tumorigenesis induced by YTHDF1 is dependent on iron metabolism and regulates transferrin receptor protein (TFRC) expression in this cancer (Ye et al., 2020). Regarding the molecular mechanism, YTHDF1 binds to the UTR of TFRC mRNA to regulate mRNA translation of TFRC (Ye et al., 2020). Targeting TFRC-mediated iron metabolism and YTHDF1 could become potential candidates for early diagnosis or treatment for HPSCC patients (Ye et al., 2020).

## Control of M^6^A by Metabolites in Cancer

In cancer, metabolism is often regulated by the m^6^A modification. But could certain metabolites regulate m^6^A? This controversial idea is supported by the finding that proteins that regulate m^6^A associate highly with many types of cancer. Also, Wang et al. (2020b) showed that nicotinamide adenine dinucleotide phosphate (NADP) binds to FTO, decreases m^6^A methylation in RNA, and promotes adipogenesis. Furthermore, NADP regulated mRNA m6A *via* FTO *in vivo*, and deletion of FTO blocked adipogenesis caused by enhanced NADP in 3T3-L1 pre-adipocytes.

Succinate prevents *α*-ketoglutarate-dependent dioxygenase from regulating critical factors of tumorigenesis, including hypoxia responses and histone demethylation. Additionally, hypoxia in tumors broadly increases levels of m^6^A in GLUT1 and MYC mRNAs (Priolo et al., 2014). ALKBH5 and FTO m^6^A demethylases require *α*-KG, Fe(II), and O_2_ for total enzymatic activity (Zhang et al., 2019; Xu and Bochtler, 2020; Losman et al., 2020). The TCA cycle produces other metabolites that regulate m^6^A demethylation. Interestingly, citrate, another critical metabolite in the TCA, was noticed with an *α*-KG-binding site in ALKBH5 (Feng et al., 2014). Citrate by binding to *α*-KG/FTO complex can inhibit the enzyme’s activity (Aik et al., 2013).

In AML cells, the FTO’s enzymatic activity is inhibited, carrying the IDH (isocitrate dehydrogenase) mutation, which correlates with significantly increased m^6^A levels (Li et al., 2017b). IDHs are critical enzymes that catalyze isocitrate to *α*-ketoglutarate (*α*-KG) and CO_2_ in the TCA cycle. They also epigenetically control gene expression through effects on *α*-KG-dependent dioxygenases. R-2HG was recently reported to exhibit antitumor activity. It attenuates aerobic glycolysis and downregulates the expression of FTO/LDHB/PFKP in leukemia cells (Qing et al., 2021). Moreover, it increases m^6^A modification of RNA by inhibiting FTO activity, destabilizing CEBPA/MYC transcripts in leukemia cells (Su et al., 2018). These findings, therefore, indicate that certain metabolites can drive the m^6^A modification of RNA in cancer (Table 2).

**TABLE 2:** Control of m^6^A by metabolites in cancer.

m^6^A implicated proteins	Metabolites	Effects	References
FTO	NADP	NADP decreases m^6^A methylation in RNA and promotes adipogenesis	Wang et al. (2020b)
FTO	R-2HG	R-2HG attenuates aerobic glycolysis and downregulates the expression of FTO in leukemia cells	Qing et al. (2021)
FTO	R-2HG	R-2HG increases m^6^A modification of RNA by inhibiting FTO activity, destabilizing MYC transcripts in leukemia cells	Su et al. (2018)
FTO	Isocitrate	Isocitrate increases m^6^A levels of RNA by inhibiting FTO’s activity in leukemia cells	Li et al. (2017b)

### Potential Clinical Applications of M^6^A and Targeting the Modification in Cancer

As proteins that create, erase and recognize m^6^A play a role in cancer metabolism, targeting altered metabolic pathways by focusing on m^6^A modification has become a promising anticancer strategy. Survival analysis of patients showed that METTL3 (a writer) is a prognostic factor for poor outcomes in HCC (Lin et al., 2020), thyroid carcinoma (Wang et al., 2020a), pancreatic cancer (Xia et al., 2019), CRC (Li et al., 2019b), gastric cancer (Wang et al., 2020c), and colorectal cancer (Chen et al., 2021a). WTAP (another writer) predicts the survival of patients with high-grade serous ovarian carcinoma (Yu et al., 2019), HCC (Chen et al., 2019c), RCC, and GC (Li et al., 2020a). As METTL3 depletion can decline oncogenes’ expression and reduce CRC proliferation (Shen et al., 2020), breast cancer (Li et al., 2020c), cervical cancer (Li et al., 2020c), and liver cancer (Wang et al., 2020c), METTL3 offers an alternative therapeutic target in colorectal cancer patients with high glucose levels (Shen et al., 2020). It also could promote colorectal tumorigenesis *via* the m^6^A-GLUT1-mTORC1 axis. Combined targeting of METTL3 and mTORC1 showed promise for suppressing CRC proliferation, suggesting that METTL3 could also be an alternative therapeutic target in that disease (Chen et al., 2021a). Deleting METTL3 from HeLa cells also decreased PDK4 expression and increased the cells’ sensitivity to doxorubicin (DOX) treatment (Li et al., 2020c). However, ectopic overexpression of PDK4 attenuated this effect and reduced DOX sensitivity in cervical cancer cells. This suggests that PDK4 is involved in the proliferation and chemosensitivity of METTL3-cells (Li et al., 2020c). Moreover, METTL3-silenced pancreatic cancer cells and glioma stem cells (GSCs) showed enhanced irradiation sensitivity (Visvanathan et al., 2018) (Taketo et al., 2018). High level of R-2HG expressed by mutant isocitrate dehydrogenase, was demonstrated to play important antitumor effect in glioma and leukemia cells by inhibiting FTO activity (Su et al., 2018).

Recently, Yankova et al. (2021) showed that STM2457, the small-molecule inhibitor targeting METTL3, might be a strategy for treating myeloid leukemia. Pharmacological METTL3 inhibition prolonged survival in AML mouse models (Yankova et al., 2021). Intriguingly, treating tumors with STM2457 increased apoptosis and reduced AML growth (Yankova et al., 2021). These results identified METTL3 inhibition as a promising therapeutic strategy for AML treatment and demonstrated that targeting enzymes that modify RNA is a new approach promising anticancer therapy (Yankova et al., 2021). Depleting METTL3 from cells induced resistance to cisplatin, gemcitabine, and 5-fluorouracil in pancreatic cancer and non-small cell lung cancer (Jin et al., 2019). Also, FTO inhibitors (FB23 and FB23-2) provide a therapeutic strategy for treating leukemia. Targeting regulators of RNA methylation have also shown promise in preclinical models, which are effective against AML, as exemplified by FB23 and FB23-2 (small-molecule inhibitors) of the m^6^A eraser FTO (Huang et al., 2019).

By pharmacological approaches, FTO is broadly viewed as an attractive biological target. Peng et al. (2019) found a small molecular inhibitor of FTO and selected m6A demethylase FTO as a potential target by developing a new strategy. By studying the molecular function of FTO in metabolism, they identified entacapone (FDA-approved drug) as a selective inhibitor of FTO activity involved in the regulation of metabolic homeostasis (Peng et al., 2019). Entacapone bound to FTO and inhibited FTO activity. They conclude that the FTO-entacapone complex may be promising for designing new drug-like FTO inhibitors as translational medicine (Peng et al., 2019). Furthermore, they discovered that the transcription factor forkhead box protein O1 (FOXO1) mRNA as a substrate of FTO, which Knockdown of FOXO1 through the inhibition of FTO could be used to treat metabolic dysregulation (Peng et al., 2019).

Targeting YTHDF1 (a reader) might be another promising therapeutic approach, as (Liu et al., 2020) identified the YTHDF1-EIF3C axis as a critical translational factor involved in ovarian cancer progression (Liu et al., 2020). Chen and collaborators (2021) recently reported that YTHDF1 is associated positively with cisplatin resistance in colon cancer (Chen et al., 2021a; Chen et al., 2021b). Furthermore, inhibition of GLS1 synergized with cisplatin to induce cell death of colon cancer cells (Chen et al., 2021b). Recently, Kumar et al., 2021 reviewed how components of EEE (Editor/Eraser/Effector) could become potential candidates for treating leukemia (Kumar et al., 2021).

Regarding immunotherapy against cancer cells, FTO was identified as an essential regulator of glycolytic metabolism that tumors could use to escape immune surveillance (Liu et al., 2021). Consistent with this idea, depleting FTO impaired the glycolytic activity of tumor cells to restore the CD8^+^ T cell function needed to inhibit tumor growth (Liu et al., 2021). Moreover, Dac51 (a small molecule) can block FTO-mediated immune evasion and control immunity, suggesting that RNA epitranscriptome could promise a new strategy for immunotherapy against cancer cells (Liu et al., 2021).

On the other hand, Yang et al. (2019) demonstrate that the effect of FTO knockdown on melanoma response to anti-PD-1 (a novel immunotherapies for the patient with melanomas) immunotherapy is dependent on the immune system. The combination of m^6^A demethylase FTO inhibition with anti-PD-1 blockade may reduce the resistance to immunotherapy in melanoma (Yang et al., 2019). Additionally, FTO depletion sensitizes melanoma cells to interferon-gamma (IFNγ) and sensitizes melanoma to anti-PD-1 treatment (Yang et al., 2019). Their findings suggest a crucial role of FTO, which increases FTO’s level, decreases response to anti-PD-1 blockade immunotherapy, and enhances tumor growth in melanoma (Yang et al., 2019). One other recent study demonstrates that the YTHDF1 reader regulated antitumor immunity, a synergetic effect on immunotherapy by improving the therapeutic effect of PD-L1 inhibitors (Han et al., 2019). Yan and collaborators demonstrate that FTO-m^6^A axis deregulation induces response to tyrosine kinase inhibitor (TKI) treatment in leukemia cells (Yan et al., 2018). Cells with FTO upregulation have more TKI tolerance and higher growth rates in mice (Yan et al., 2018). Currently, Li and collaborators demonstrated that the JPX/FTO/PDK1 axis could facilitate aerobic glycolysis in GBM cells, which was correlated with GBM cells’ sensitivity to temozolomide (TMZ). These findings provide valuable information for understanding that blocking the JPX/FTO/PDK1 axis may serve as a promising strategy for mitigating the efficacy of TMZ in GBM(Li et al., 2021).

By elucidating the biological roles of m^6^A’s modification in natural killer (NK) cells, Ma and collaborators uncovered a new direction for harnessing NK Cell antitumor immunity. YTHDF2 deficiency in NK Cells impaired NK Cells’ antitumor and antiviral activity *in vivo*. Upon activation by cytokines, YTHDF2 is upregulated in NK Cells. More interestingly, YTHDF2 promoted NK Cell effector function by inhibiting a STAT5-YTHDF2-positive feedback loop involved in tumor progression (Ma et al., 2021). These findings suggested that m^6^A and its regulatory or associated proteins are involved in cancer progression. The development of new applicable inhibitors or the translation of existing inhibitors into clinical practice may provide innovative and effective therapeutic strategies for treatment (Table 3).

**TABLE 3:** Non-exhaustive list of Potential alternative therapeutic agents offers by m^6^A targeting modifications in cancer.

m^6^A proteins involved	Drugs/Therapeutic agents	Metabolites Pathways/Immune system	Underlying mechanism and Key results	References
METTL3	Doxorubicin (DOX)	Glycolytic metabolism/Antitumor	METTL3 depletion decreased PDK4 expression and increased sensitivity to doxorubicin treatment in cervical cancer cells	Li et al. (2020c)
METTL3	STM2457	Antitumor	STM2457 by targeting METTL3 increased apoptosis and reduced AML growth treating myeloid leukemia	Yankova et al. (2021)
METTL3	Cisplatin, Gemcitabine, 5-fluorouracil	Antitumor	Depleting METTL3 from cells induced resistance to cisplatin, gemcitabine, and 5-fluorouracil in pancreatic cancer and non-small cell lung cancer	Jin et al. (2019)
METTL3	Gamma-irradiation	Antitumor	METTL3-silenced pancreatic cancer cells and glioma stem cells showed enhanced irradiation sensitivity	Visvanathan et al. (2018)
FTO	R-2HG	Metabolic regulation/ Antitumor	R-2HG, highly expressed by isocitrate dehydrogenase, inhibit FTO and act an antitumor in glioma and leukemisa cells	Su et al. (2018)
FTO	Entacapone	Metabolic regulation/Antitumor	Entacapone bound to FTO and inhibited FTO activity involved in the regulation of metabolic homeostasis and amino acid metabolism	Peng et al. (2019)
FTO	FB23 and FB23-2	Antitumor	Targeting FTO, FB23 and FB23-2 are effective promise in preclinical models against acute myeloid leukemia	Huang et al. (2019)
FTO	Tyrosine kinase inhibitor (TKI)	Immunity control	Disregulated FTO help tumor cells to escape TKI-mediated killing and broad defense mechanism by which leukemia cells develop resistance mechanism to TKI	Yan et al. (2018)
FTO	Dac51	Antitumor/Immunity control	Small molecule Dac51 can block FTO-mediated immune evasion and control immunity against cancer cells	Liu et al. (2021)
FTO	Glycolytic agents	Immunity control	Disregulated complex FTO - glycolytic agents help tumor cells to escape immune surveillance	Liu et al. (2021)
FTO	Anti-PD-1 blockade	Antitumor immunity	Knockdown of FTO sensitizes melanoma cells to interferon-gamma (IFNγ) and sensitizes melanoma to anti-PD-1 treatment in mice	Yang et al. (2019)
FTO	Temozolomide (TMZ)	Glycolytic metabolism/Antitumor	JPX/FTO/PDK1 axis facilitate aerobic glycolysis in GBM cells, and correlated with GBM cells' sensitivity to temozolomide	Li et al. (2021)
YTHDF1	Cisplatin	Amino acid metabolism/Antitumor	YTHDF1 is associated with cisplatin resistance in colon cancer.Inhibition of GLS1 synergized with cisplatin to induce cell death of colon cancer cells	Chen et al. (2021b)
YTHDF1	PD-L1 inhibitor	Antitumor immunity	YTHDF1 regulate antitumor immunity and have synergetic effect on immunotherapy by improving the therapeutic effect of PD-L1 inhibitor	Han et al. (2019)
YTHDF2	STAT5	Immune response	Upon activation by cytokines, YTHDF2 is upregulated in NK Cells. YTHDF2 promoted NK Cell effector function by inhibiting a STAT5-YTHDF2-positive feedback loop involved in tumor progression	Ma et al. (2021)

## Conclusion and Perspectives

The connection between metabolism and tumorigenesis is attracting attention, and many gratifying results have revealed the link between the m^6^A modification and oncometabolite in cancer progression. The data demonstrates that the m^6^A modification regulators could act as promising candidates for diagnosis, prognosis, or treatment against cancer. Thus, designing a diagnostic profile for cancer is possible based on oncometabolite regulated by m^6^A. In this review, the potential crosstalk between m^6^A RNA methylation and metabolic control in tumorigenesis was described. These findings build a link between metabolic reprogramming and the m^6^A modification. As investigators have focused mostly on glucose metabolism and performed *in vitro* studies with cell lines, their investigations need to be validated in animal models and clinical studies.

As integrated regulation of metabolism in cancers, the network of several major anabolic and catabolic pathways are important co-factors or substrates of the critical enzymes for RNA modifications. Since many of the metabolic alterations and consequently aberrated RNA regulation are common to a wide range of cancer types, they can serve as promising targets for anti-cancer therapies. Considering current efforts to target both cancer metabolism and regulation of the epigenome, it is still elusive to fully clarify the critical downstream factors functions mediated by some oncometabolite in cancer cells. Understanding the integrated metabolism in cancer cells may open new avenues for anti-cancer strategies. Therefore, determining metabolic differences between normal proliferating and cancer cells will be of great interest. Nevertheless, heterogeneity of tumors is yet another challenge, which is multiples phenotypes metabolic in multi-cellular systems. In addition, more researches should be conducted to better understand the molecular mechanisms among metabolic enzymes, transporters, transcription factors, and their pathways regulated by the m^6^A modification in cancer metabolism.

By pharmacological approach, evidence has shown that characterization of m^6^A writers and erasers proteins have provided valuable insights promising anti-cancer drugs targeting modification in cancer. While several small-molecule inhibitors targeting writers or erasers are either approved drugs or are currently being evaluated in clinical trials, the targeting m^6^A recognition proteins have lagged behind. After writers and erasers carry out methylation and demethylation, the readers determine the functional consequences of modification. Thus, more investigations and pharmacological research needs to target m^6^A readers in cancer progression to yield meaningful results.

Most importantly, attempts to target m^6^A pathways and their associated metabolic pathways need to consider immune cells, as m^6^A was recently reported to play roles in antitumor immunity, immune responses, and immunotherapy in cancers (Liu et al., 2021; Ma et al., 2021). Such an approach will help us better understand and fully clarify how the dysregulation of metabolism by m^6^A in tumorigenesis jeopardizes immune surveillance. As well as regulating glucose, amino acids, and lipids, m^6^A can regulate other metabolites, such as SAM, SAH, IDH, R-2HG, vitamin C, and iron. It will be interesting to understand how the m^6^A modification affects those compounds and how that knowledge could enhance cancer treatment. As m^6^A often alters metabolism, some metabolites might also regulate the production, editing, and recognition of m^6^A to affect cancer progression. Due to this controversial idea, it will also be interesting to discover how metabolite signaling networks regulate m^6^A in cancer and how they, in turn, could be regulated.
